# Thyroid cancer treatment among adolescents and young adult women and reproductive outcomes: a population-based cohort study

**DOI:** 10.1093/hropen/hoaf070

**Published:** 2025-11-13

**Authors:** Haris Imsirovic, Harriet Richardson, Jonas Shellenberger, Maria P Velez

**Affiliations:** Ottawa Hospital Research Institute, Ottawa, ON, Canada; Department of Public Health Sciences, Queen’s University, Kingston, ON, Canada; ICES, Toronto, ON, Canada; ICES, Toronto, ON, Canada; Department of Obstetrics and Gynecology, McGill University, Montreal, QC, Canada

**Keywords:** thyroid cancer, thyroidectomy, radioactive iodine, infertility, menopause, childbirth

## Abstract

**STUDY QUESTION:**

Are different types of thyroid cancer treatment among young women associated with adverse reproductive outcomes?

**SUMMARY ANSWER:**

All types of thyroid cancer treatment were associated with infertility diagnosis and early menopause, but not associated with premature ovarian insufficiency (POI) or lower childbirth rates.

**WHAT IS KNOWN ALREADY:**

Thyroid cancer and/or its treatment may affect thyroid function, and thyroid hormone imbalances may affect reproduction.

**STUDY DESIGN, SIZE, DURATION:**

Population-based matched cohort study included adolescent and young adult women (AYAs, 15–39 years) treated for thyroid cancer in Ontario, Canada between 1992 and 2019, after they had lived at least 3 years free of recurrence.

**PARTICIPANTS/MATERIALS, SETTING, METHODS:**

Each participant was matched to five cancer-free women based on age, census subdivision, parity, and year of cancer diagnosis. The study cohort consisted of 6474 women undergoing thyroid cancer treatment and 31 922 women without cancer. Exposure status was determined by thyroid cancer treatment, namely: (i) cancer-free (unexposed; referent), (ii) less than total thyroidectomy (LTT), (iii) total thyroidectomy (TOT), or (iv) total thyroidectomy in combination with radioactive iodine therapy (TOT+RAI). Among the exposed, 3396 (52.5%) received TOT, 1520 had (23.5%) LTT, and 1558 (24.1%) had TOT+RAI. Our main outcomes of interest were infertility diagnosis, POI (i.e. cessation of ovarian function before age 40 years), early menopause (menopause before age 45 years), and childbirth rates. Poisson regression models generated weighted relative risks (wRR) using inverse probability of treatment weighting to adjust for imbalances in baseline characteristics.

**MAIN RESULTS AND THE ROLE OF CHANCE:**

Mean (SD) age at thyroid cancer diagnosis was 30.6 (6.1) years. The rate of infertility was 3186/31 922 (10.0%) among unexposed, 177/1520 (11.6%) after LTT, 414/3396 (12.2%) after TOT, and 213/1558 (13.7%) following TOT+RAI. The weighted relative risk (wRR) was 1.26 (1.12–1.39) for LTT, 1.22 (1.13–1.32) for TOT, and 1.34 (1.19–1.48) following TOT+RAI. The rate of early menopause was 713/31 922 (2.2%) among unexposed, 46/1520 (3.0%) after LTT, 78/3396 (2.3%) after TOT, and 54/1558 (3.5%) following TOT+RAI. The wRR was 1.42 (1.09–1.72) for LTT, 1.02 (0.83–1.20) for TOT, and 1.54 (1.21–1.89) following TOT+RAI. The rates of POI and childbirth were similar between the unexposed and treatment groups.

**LIMITATIONS, REASONS FOR CAUTION:**

Misclassification is a possibility when using linkage of administrative databases. Absence of information about thyroid hormone supplementation and TSH levels in the study databases is another limitation. Since the unexposed group consisted of cancer-free women, we cannot distinguish whether the observed associations reflect the effects of thyroid cancer itself or its treatment.

**WIDER IMPLICATIONS OF THE FINDINGS:**

In this study, thyroid cancer and/or all types of its treatment were associated with a higher rate of infertility diagnosis and early menopause, but not associated with POI or lower childbirth rates. These findings highlight the need for counselling and surveillance about reproductive outcomes among AYA women with thyroid cancer.

**STUDY FUNDING/COMPETING INTEREST(S):**

This work was supported by the Research Institute McGill University Health Centre and a Fonds de recherche du Québec—Santé (FRQ-S) Chercheur Boursier Clinicien award. All of the authors have no conflicts of interest to declare.

**TRIAL REGISTRATION NUMBER:**

N/A.

WHAT DOES THIS MEAN FOR PATIENTS? As thyroid cancer and/or its treatments may affect thyroid function, and thyroid hormone imbalances may affect reproduction, we conducted a population-based matched cohort study to determine whether different types of thyroid cancer treatment among young women are associated with adverse reproductive outcomes. The results showed that all young women treated for thyroid cancer may face a higher chance of being diagnosed with infertility and may experience menopause at an earlier age. However, childbirth rates do not appear to be reduced. These findings may guide patients with thyroid cancer to make informed decisions about fertility and family planning and encourage them to seek counselling if concerned about their reproductive health.

## Introduction

The incidence of thyroid cancer is increasing, which is in part explained by an increase of surveillance and new diagnostic technologies that result in early diagnosis (Nuttall *et al.*, 2017; Topstad and Dickinson, 2017; Brenner *et al.*, 2024). Thyroid cancer is mainly treated with surgery, including less than total thyroidectomy (LTT) or total thyroidectomy (TOT), or surgery in combination with radioactive iodine therapy (RAI) (PDQ Adult Treatment Editorial Board, 2024). Normal thyroid function is important for reproduction (Krassas *et al.*, 2010). In women, the peak of thyroid cancer incidence occurs most often during the reproductive years (Sakoda and Horn-Ross, 2002), raising concern about the potential impact of thyroid cancer and its treatments on reproductive outcomes (Albritton *et al.*, 2006). Impacts on fertility potential is known to have a major impact on the quality of life of adolescent and young adult (AYA) cancer survivors (Rosen *et al.*, 2009), defined by the U.S. National Cancer Institute to be between the ages of 15 and 39 years (Albritton *et al.*, 2006). Numerous studies have reported that there are very low rates of discussion between patients and health care providers regarding the potential gonadotoxic effects of cancer treatment on fertility (Duffy *et al.*, 2005; Quinn *et al.*, 2007, 2009; Wallace, 2007; Yee *et al.*, 2012). Of greater concern, a significant proportion of the dialogue is prompted by the patients rather than the health care providers (Ah, 2004). Therefore, it is crucial to assess the relative effect of different thyroid cancer treatment options on reproductive outcomes.

We have identified an increased risk of infertility and premature ovarian insufficiency (POI) after thyroid cancer diagnosis among AYAs, however, knowledge of the role of different modalities of thyroid cancer treatment on reproductive outcomes is limited (Velez *et al.*, 2021; Flatt *et al.*, 2023). Some studies suggest that surgical treatment for thyroid cancer is not associated with adverse reproductive outcomes if TSH levels are maintained within normal limits (Rahman *et al.*, 2010; Kim *et al.*, 2011; Verma *et al.*, 2012; Jefferys *et al.*, 2015). However, a retrospective cohort study examining pregnancy and live birth rates of Chinese women treated for thyroid cancer and underwent IVF/ICSI cycles showed that those who underwent TOT had lower clinical pregnancy and live birth rates in comparison to those undergoing partial thyroidectomy (Huang *et al.*, 2021). A systematic review suggested that RAI therapy may result in transient amenorrhoea and earlier age at menopause, but childbirth rates were not affected (Clement *et al.*, 2015). A recent population-based study from Taiwan reported that thyroid cancer patients had a higher incidence rate of infertility compared to controls, and this was not associated with RAI administration (Lin *et al.*, 2025). The impact of surgery alone versus the combination of surgery and RAI on female reproductive outcomes needs further investigation.

To address clinically relevant endpoints across the reproductive window, we conducted a population-based matched cohort study within a public health care system to evaluate the association between type of thyroid cancer treatment and four outcomes of interest: (i) infertility diagnosis, as a healthcare-seeking proxy for difficulty conceiving; (ii) POI, defined as menopause before age 40 years; (iii) early menopause, defined as menopause between the ages of 40 and 45 years; and (iv) childbirth, capturing realized fertility. Prior literature has variably considered surgery and RAI, but their comparative effects remain uncertain. Accordingly, our objective was to evaluate comparative risks across treatment modalities: LTT, TOT, TOT, and RAI. The control group consisted of matched cancer-free women.

## Materials and methods

This retrospective population-based matched study was conducted using existing linked administrative health data from Ontario, Canada (Supplementary Table S1). Data were linked using unique identifiers and analysed at ICES, an independent, non-profit research institute whose legal status under Ontario’s health information privacy law allows it to collect and analyse health care and demographic data, without consent, for health system evaluation and improvement. The study followed the STROBE reporting guideline for cohort studies and was reviewed for ethical compliance by the Institutional Research Ethics Board.

### Data sources and study cohort creation

This study comprises all women in Ontario with a diagnosis of thyroid cancer between 1 January 1992 and 31 December 2019, aged 15–39 years, identified through the Ontario Cancer Registry (OCR). For cancer-free controls, we assigned a pseudo-index date sampled from the distribution of diagnosis dates in the exposed cohort. This alignment of calendar time reduces confounding from temporal trends in coding practices, fertility care, and thyroid cancer management. Each woman was matched to five cancer-free women by age, census subdivision, parity, and year of cancer diagnosis. Parity was measured prior to index and used in matching to control for strong confounding by prior reproductive history and preferences, which are associated with both infertility diagnosis and childbirth (Moreau *et al.*, 2010; Chambers *et al.*, 2020). However, given some exclusions applied after the matching was conducted, 4.6% of exposed women were matched to three (N = 150) or four (N = 149) women. Thyroid treatment type was obtained from the Discharge Abstract Database (DAD) and National Ambulatory Care Reporting System (NACRS). Demographic information, including sex, age, income, and rurality status was obtained using the Registered Persons Database (RPDB) and the Postal Code Conversion File (PCCF). Immigration information was captured using the Immigration, Refugee and Citizenship Canada Permanent Resident (IRCC-PR) database. Parity status was determined using the MOMBABY database. Study outcomes were identified through the Ontario Health Insurance Plan (OHIP) claims database.

Excluded were women who did not receive treatment within 2 years after thyroid cancer diagnosis, those who died within 3 years of cancer diagnosis, and those with under 3 years of continuous valid OHIP coverage. Women with prior cancer diagnosis, or a new primary cancer diagnosis within 1 year after thyroid cancer were also excluded, as were those with thyroid cancer recurrence within 3 years. Due to the rarity of using RAI as a standalone treatment (Cooper *et al.*, 2009), patients who only received RAI for treatment of thyroid cancer were not eligible. Similarly, since it is not recommended to ablate the remaining lobe with RAI after receiving LTT, those patients who had LTT + RAI treatment were also excluded (Cooper *et al.*, 2009). Excluded were also women with a history of hysterectomy, bilateral oophorectomy, and/or tubal ligation at any point up to 3 years after cancer treatment. Those who received a diagnosis of infertility, POI or early menopause prior to thyroid cancer treatment or within the first year were also excluded as a temporal association with cancer treatment could not be confirmed.

### Exposure

Exposure status was by type of thyroid cancer treatment, namely: (i) absence of cancer (unexposed; referent), (ii) LTT, (iii) TOT, or (iv) total thyroidectomy in combination with radioactive iodine therapy (TOT+RAI).

### Outcomes

Study outcomes were infertility diagnosis, POI, early menopause, and childbirth. Infertility diagnosis was identified using information on claims billed by physicians through the universal OHIP database (ICD-9 628) starting after 1 year of thyroid cancer treatment. POI was defined as a menopause diagnosis (ICD-9 627) before age 40 years, and early menopause as a menopause diagnosis (ICD-9 627) between ages 40 and 45 years. Childbirth was defined as the presence of a live or stillborn >20 weeks gestation in the MOMBABY database.

### Covariates

Covariates known to be associated with thyroid cancer treatment and/or reproductive outcomes were selected *a priori* and included income status, rurality, immigration status, prior endometriosis, prior PCOS, smoking status, obesity, and comorbidities. If imbalances remained post-matching, matching variables (age, parity) were adjusted for as well. Comorbidities were identified using ICD-9 and ICD-10 codes through an algorithm developed by Mondor *et al.* (Koné Pefoyo *et al.*, 2015; Lane *et al.*, 2015; Gruneir *et al.*, 2016; Mondor *et al.*, 2016, 2017, 2018; Petrosyan *et al.*, 2017; Thavorn *et al.*, 2017; Rosella *et al.*, 2018).

### Statistical analyses

Descriptive statistics for baseline sociodemographic and medical conditions were compared across exposure groups using standardized differences, with a difference of more than 0.1 suggesting a meaningful difference. Logistic regression was used to estimate probability of assignment to each exposure level conditional on the covariates listed above, and these probabilities were used to compute pairwise inverse probability of treatment weights targeting the average treatment effect in the treated (ATT). We then used Poisson regression models on the weighted cohort to estimate the ATT as rate ratios. Women were followed-up until outcome occurrence, 31 December 2022, or death. Analyses assessing POI were restricted to patients who were under 39 years of age at thyroid cancer diagnosis as POI is defined as the cessation of ovarian function before the age of 40 years.

All analyses were completed using SAS software v9.4 (SAS Institute Inc. Cary, NC, USA).

## Results

**Figure 1.**
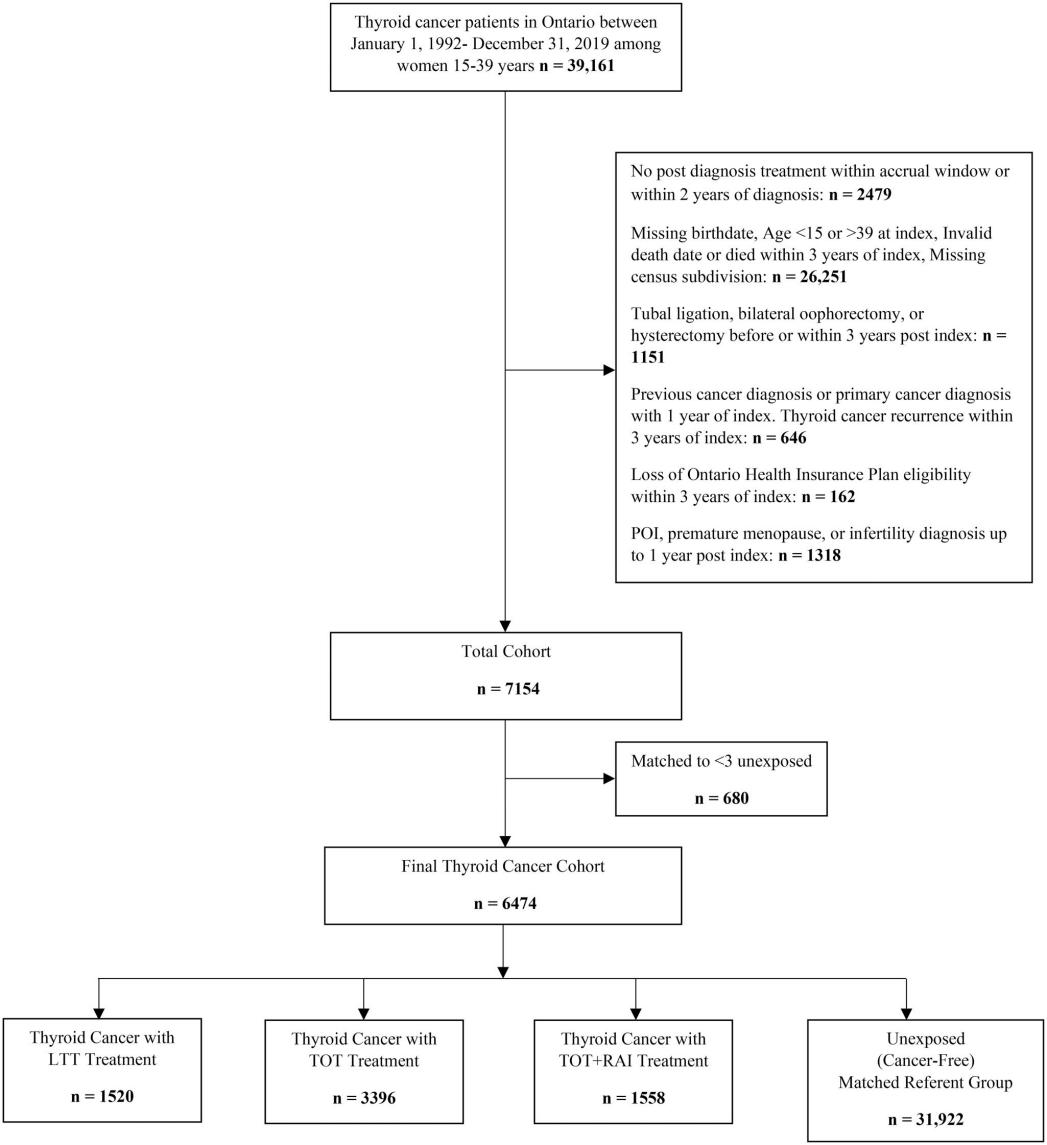
**Flow chart of cohort creation.** LTT, less than total thyroidectomy; TOT, total thyroidectomy; TOT+RAI, total thyroidectomy in combination with radioactive iodine therapy.

A total of 6474 women treated for thyroid cancer and 31 922 women without a cancer diagnosis (referent ‘unexposed’ group) were eligible for the study (Fig. 1). Of those treated for thyroid cancer, 52.5% received TOT, 23.5% LTT, and 24.1% TOT+RAI. Table 1 summarizes baseline characteristics for the entire cohort. The cancer-free and thyroid cancer treatment groups were similar in terms of age, income status, residential rurality, parity, immigration status, smoking history, obesity, and gynaecological conditions of interest at baseline. The mean (SD) age at cohort start was 30.6 (6.1) years for unexposed, 31.0 (5.8) for LTT, 30.6 (6.2) for TOT, and 30.4 (6.2) for TOT+RAI. However, the exposed group (any treatment) was more likely to have multiple comorbidities compared to those without cancer (26% vs 20.4%; standardized difference = 0.13).

**Table 1.** Baseline characteristics by type of exposure among women living in Ontario, Canada between 1 January 1992 and 31 December 2019.

Characteristics	Unexposed	LTT	TOT	TOT+RAI	Standardized difference	Standardized difference	Standardized difference
	N = 31 922	N = 1520	N = 3396	N = 1558	LTT vs unexposed	TOT vs unexposed	TOT + RAI vs unexposed
**Age at Index, mean (SD)**	30.6 (6.1)	31.0 (5.8)	30.6 (6.2)	30.4 (6.2)	0.07	0.01	0.04
**Rural residence**	153 (0.5)	22 (0.6)	10 (0.6)	7 (0.5)	0.00	0.02	0.02
**Income Quintile**							
Lowest Quintile	7134 (22.3)	327 (21.5)	649 (19.1)	345 (22.1)	0.02	0.08	0.01
Second Quintile	6494 (20.3)	329 (21.6)	698 (20.6)	300 (19.3)	0.03	0.01	0.03
Middle Quintile	6366 (20.0)	318 (21.0)	665 (19.6)	332 (21.3)	0.02	0.01	0.03
Fourth Quintile	6145 (19.3)	301 (19.8)	687 (20.2)	334 (21.4)	0.01	0.03	0.05
Highest Quintile	5783 (18.1)	245 (16.1)	697 (20.5)	247 (15.9)	0.05	0.06	0.06
**Immigrants**	9683 (30.3)	448 (29.5)	932 (27.4)	540 (34.7)	0.02	0.06	0.09
**Parous**	8548 (26.8)	420 (27.6)	912 (26.9)	453 (29.1)	0.02	0.00	0.05
**Endometriosis**	334 (1.0)	23 (1.5)	49 (1.4)	20 (1.3)	0.04	0.04	0.02
**Polycystic Ovary Syndrome**	663 (2.1)	59 (3.9)	117 (3.4)	59 (3.8)	0.11	0.08	0.10
**Smoking history**	673 (2.1)	28 (1.8)	63 (1.9)	20 (1.3)	0.02	0.02	0.06
**Obesity**	3820 (12.0)	210 (13.8)	482 (14.2)	253 (16.2)	0.06	0.07	0.12
**Comorbidities**							
None	15 407 (48.3)	605 (39.8)	1384 (40.8)	652 (41.8)	0.17	0.15	0.13
One	9987 (31.3)	532 (35.0)	1111 (32.7)	506 (32.5)	0.08	0.03	0.03
Two or more	6528 (20.4)	383 (25.2)	901 (26.5)	400 (25.7)	0.11	0.14	0.12

Data are N (%) unless otherwise stated. A standardized difference of more than 0.1 suggests a meaningful difference. LTT, less than total thyroidectomy; TOT, total thyroidectomy; TOT+RAI, total thyroidectomy in combination with radioactive iodine therapy.

Women were followed-up for a median (IQR) of 9 (5–15) years when assessing infertility diagnosis. Median follow-up across exposure groups was 9 (5–15) years for unexposed, 7 (4–14) years for LTT, 8 (5–14) years for TOT, and 10 (6–15) years for TOT+RAI. The rate of infertility was 10.0% among unexposed, 11.6% after LTT, 12.2% after TOT, and 13.7% following TOT+RAI. The weighted relative risk (wRR) for infertility diagnosis was 1.26 (95% CI, 1.12–1.39) for LTT, 1.22 (95% CI, 1.13–1.32) for TOT, and 1.34 (1.19–1.48) following TOT+RAI (Table 2).

**Table 2.** Association between thyroid cancer treatment and risk of adverse reproductive outcomes.

Outcome	No. with outcome/No. at risk	Crude rate (%)	Unadjusted relative risk	Weighted relative risk
**Infertility diagnosis**				
Unexposed	3186/31 922	9.98	1.00 (Ref.)	1.00 (Ref.)
LTT	177/1520	11.64	1.30 (1.16–1.43)	1.26 (1.12–1.39)
TOT	414/3396	12.19	1.26 (1.17–1.35)	1.22 (1.13–1.32)
TOT+RAI	213/1558	13.67	1.39 (1.24–1.54)	1.34 (1.19–1.48)
**Premature Ovarian Insufficiency** ^a^				
Unexposed	710/29 894	2.38	1.00 (Ref.)	1.00 (Ref.)
LTT	36/1417	2.54	1.22 (0.89–1.51)	1.17 (0.84–1.46)
TOT	90/3191	2.82	1.20 (0.99–1.37)	1.15 (0.96–1.32)
TOT+RAI	38/1459	2.60	1.04 (0.80–1.29)	1.00 (0.77–1.24)
**Early menopause**				
Unexposed	713/31 922	2.23	1.00 (Ref.)	1.00 (Ref.)
LTT	46/1520	3.03	1.51 (1.16–1.83)	1.42 (1.09–1.72)
TOT	78/3396	2.30	1.05 (0.84–1.24)	1.02 (0.83–1.20)
TOT+RAI	54/1558	3.47	1.56 (1.23–1.92)	1.54 (1.21–1.89)
**Childbirth**				
Unexposed	8208/31 922	25.71	1.00 (Ref.)	1.00 (Ref.)
LTT	376/1520	24.74	1.07 (0.98–1.15)	1.07 (0.99–1.17)
TOT	902/3396	26.56	1.05 (1.00–1.11)	1.06 (1.01–1.11)
TOT+RAI	480/1558	30.81	1.26 (1.18–1.35)	1.22 (1.15–1.29)

Weighted relative risks are adjusted for age, income status, rurality, immigration status, parity, prior endometriosis, prior PCOS, smoking status, obesity, and comorbidities. LTT, less than total thyroidectomy; TOT, total thyroidectomy; TOT+RAI, total thyroidectomy in combination with radioactive iodine therapy.

a Analyses restricted to patients who were under 39 years of age at thyroid cancer diagnosis.

Women were followed-up for a median (IQR) of 5 (3–9) years when assessing POI diagnosis. Median follow-up across exposure groups was 5 (3–9) years for unexposed, 5 (3–8) years for LTT, 5 (3–9) years for TOT, and 6 (3–10) years for TOT+RAI. The rate of POI was 2.4% among unexposed women, 2.5% after LTT, 2.8% after TOT, and 2.6% following TOT+RAI. The wRR for POI was 1.17 (95% CI, 0.84–1.46) for LTT, 1.15 (95% CI, 0.96–1.32) for TOT, and 1.00 (0.77–1.24) following TOT+RAI (Table 2).

Women were followed-up for a median (IQR) of 10 (6–15) years when assessing early menopause. Median follow-up across exposure groups was 10 (6–16) years for unexposed, 8 (4–14) years for LTT, 9 (6–15) years for TOT, and 11 (7–15) years for TOT+RAI. The rate of early menopause was 2.2% among unexposed women, 3.0% after LTT, 2.3% after TOT, and 3.5% following TOT+RAI. The wRR for early menopause was 1.42 (95% CI, 1.09–1.72) for LTT, 1.02 (95% CI, 0.83–1.20) for TOT, and 1.54 (1.21–1.89) following TOT+RAI (Table 2).

Women were followed-up for a median (IQR) of 7 (4–13) years when assessing childbirth after cohort start. Median follow-up across exposure groups was 7 (4–13) years for unexposed, 6 (3–12) years for LTT, 7 (4–12) years for TOT, and 8 (4–13) years for TOT+RAI. The rate of childbirth was 25.7% among unexposed women, 24.7% after LTT, 26.6% after TOT, and 30.8% following TOT+RAI. The wRR for childbirth was 1.07 (95% CI, 0.99–1.17) for LTT, 1.06 (95% CI, 1.01–1.11) for TOT, and 1.22 (1.15–1.29) following TOT+RAI (Table 2).

## Discussion

This study suggests that thyroid cancer and/or its treatment may be associated with subsequent infertility diagnosis and early menopause. However, no association was observed for POI or lower childbirth rates.

Limited studies have assessed reproductive outcomes after thyroid cancer treatment. In relation to infertility, contrary to our findings, a recent population-based study from Taiwan reported no association between RAI treatment and risk of infertility, however, the different modalities of surgical treatment were not considered in the study (Lin *et al.*, 2025).

This study did not find an association between thyroid cancer treatment and POI (cessation of ovarian function before age 40 years), which is different to our prior study where women with thyroid cancer diagnosis had an increased risk of POI (RR 1.26, 95% CI 1.09–1.46) (Flatt *et al.*, 2023). However, the proportions of patients with POI were very similar in both studies (2.8% in Flatt *et al.*, vs 2.7% current study). An important difference between Flatt *et al.*, and this study, is that the former included all patients with thyroid cancer independent of surgical or RAI treatment. Both the 2015 American Thyroid Association (ATA) and the 2018 National Comprehensive Cancer Network (NCCN) guidelines suggest active surveillance as a management option for certain low-risk thyroid cancers (Haugen *et al.*, 2016; Haddad *et al.*, 2018). A 2020 US population study projected that, by 2025, 23.8%–31.7% of newly diagnosed thyroid cancer patients would be potential candidates for active surveillance as a treatment strategy (Roman *et al.*, 2020). This, as well as differences in analytical approaches (different follow-up windows, covariate control) could in part explain the absence of association between thyroid cancer treatment and POI in the current study. Future studies are needed in relation to potential mechanistic pathways between thyroid cancer and the risk of POI, independent of treatment. Nonetheless, in this study, thyroid cancer treatment was associated with early menopause (menopause between 40 and 45 years), a finding that was reported in a prior propensity-score matched study (Clement *et al.*, 2015).

We observed an elevated risk of early menopause after LTT and after TOT+RAI, but not after TOT. Following TOT, patients typically receive full levothyroxine replacement with tighter TSH titration, potentially stabilizing thyroid–gonadal signalling (Haugen *et al.*, 2016; Silva *et al.*, 2018; Brown *et al.*, 2023). By contrast, residual thyroid tissue after LTT can lead to periods of subclinical hypothyroidism or iatrogenic hypothyroidism during dose adjustment, perturbing gonadotropin dynamic and menstrual cyclicity (Haugen *et al.*, 2016; Silva *et al.*, 2018; Brown *et al.*, 2023). TOT+RAI likely confers additional, gonad-independent ovarian effects from radioiodine. Low-to-moderate doses to the ovaries are consistently associated with declines in serum anti-Müllerian hormone after RAI (Evranos *et al.*, 2018; Kitahara *et al.*, 2019; van Velsen *et al.*, 2020; Anagnostis *et al.*, 2021; Piek *et al.*, 2021). Several studies have reported menstrual irregularities in the first-year post-RAI (Sioka *et al.*, 2006; Piek *et al.*, 2021). However, the effects are transient (Evranos *et al.*, 2018). Further research is needed to understand this potential effect.

Despite an increased risk of infertility diagnosis, women treated for thyroid cancer had similar and, in some instances, higher rates of childbirth. This apparent paradox can be reconciled by recognizing that infertility diagnosis is a care-seeking and coding outcome, whereas childbirth captures realized fertility (Oktay *et al.*, 2018). Increased medical surveillance following thyroid cancer diagnosis and treatment could lead to increased opportunities for family-building discussions, screenings for subfertility, and earlier referral to fertility care. In our study, childbirth is ascertained through vital records (MOMBABY), which is less susceptible to surveillance bias than diagnosis codes used for infertility. Moreover, clinical guidance recommends deferring conception for 6–12 months after RAI and until thyroid hormone replacement and TSH targets are stabilized after thyroidectomy (Haugen *et al.*, 2016). This can lengthen time-to-pregnancy without reducing the cumulative probability of childbirth over multi-year follow-up. Studies in thyroid cancer survivors show longer times to first pregnancy but no overall decrease in first-birth rates after RAI (Wu *et al.*, 2015; Anderson *et al.*, 2017; Hirsch *et al.*, 2023). This is consistent with the results of our study, suggesting that fertility is deferred rather than diminished overall. Additionally, motivational and behavioural factors likely matter. Patients who survive thyroid cancer may prioritize family planning post-treatment, leading to higher rates of ART use or changes in reproductive decision-making. In survivorship cohorts, ART utilization and success rates are broadly comparable to that of controls when access is available, supporting the idea that proactive care can offset subfertility (Keefe *et al.*, 2024). Similar results were shown in other cancer populations, where despite fertility concerns, increased understanding and proactive reproductive planning contributed to an increase in childbirth rates (Wallace, 2007; Jeruss and Woodruff, 2009).

Among the strengths of this study are its population-based design and analytical approach, however, we acknowledge some limitations. Use of the billing code for female infertility (ICD-9 628) utilized in this study to identify the cohort of women with infertility has not been validated. However, The Massachusetts Outcome Study of Assisted Reproductive Technology (MOSART) cohort in Massachusetts, USA used a similar approach to identify women with infertility who did not require Assisted Reproductive Technologies to conceive (Luke *et al.*, 2016). Also, the study by Ko *et al.* used the ICD-9 628 code to control for infertility in their analysis (Ko *et al.*, 2016), and a study by Jensen *et al.* used the ICD-9 code 628 as one of the diagnosis criteria for infertility (Jensen *et al.*, 2007). We acknowledge that our study only identifies women who presented seeking care for infertility and would not capture those who experienced infertility but did not seek medical assistance. Similarly, the billing code (ICD-9 627) used to identify POI and early menopause has not been validated either. Flatt *et al.* showed that the use of the ICD-9 627 code as diagnosis of POI resulted in low sensitivity (30.1%), but high specificity (97.0%) when validated against FSH levels >25 IU/L (Flatt *et al.*, 2023). Therefore, under-ascertainment and misclassification of reproductive outcomes is a possibility in this study. However, such misclassification is likely to bias the results towards the null. Absence of information about thyroid hormone supplementation and TSH levels in the study databases is another limitation. Additionally, because the control group included cancer-free women, we cannot separate the effects of thyroid cancer itself from those of its treatments. Residual confounding related to diagnosis, surveillance, or other cancer-related factors cannot be excluded. Finally, we lacked data on histological subtype of thyroid cancer. Since histology can influence treatment decisions, such as use of RAI, it may act as a confounder in the relationship between treatment and reproductive outcomes. The inability to adjust for histology in our propensity score modelling or outcome analyses raises the possibility of residual confounding if reproductive risks differ by tumour subtype. Future studies with access to detailed histopathologic data will be needed to clarify whether treatment effects vary according to underlying tumour biology.

In conclusion, although thyroid cancer and/or its treatment among AYAs may be associated with higher rates of infertility diagnosis, childbirth rates seem not to be affected. While the rate of POI was similar across the exposure groups, thyroid cancer and/or its treatment were associated with higher rates of early menopause. These findings provide new insight on the impact of different types of thyroid cancer treatment on reproductive outcomes and highlight a need for further research to discern potential mechanisms for these associations. Furthermore, results of this study can inform patient counselling about the potential association between different types of thyroid cancer treatment and reproductive outcomes.

## Supplementary Material

hoaf070_Supplementary_Data

## Data Availability

The data set from this study is held securely in coded form at ICES. While data-sharing agreements prohibit ICES from making the data set publicly available, access may be granted to those who meet prespecified criteria for confidential access, available at www.ices.on.ca/DAS. The full data set creation plan and underlying analytic code are available from the authors upon request, understanding that the computer programmes may rely upon coding templates or macros that are unique to ICES and therefore either inaccessible or requiring modification.
