# Supplementary material for: Thyroid cancer treatment among adolescents and young adult women and reproductive outcomes: a population-based cohort study
Source: Hum Reprod Open. 2025 Nov 13;2025(4):hoaf070. doi: 10.1093/hropen/hoaf070 (PMC12671970; doi:10.1093/hropen/hoaf070)
Supplement: hoaf070_Supplementary_Data [file hoaf070_supplementary_data.docx]

**Supplemental Tables:**

**Supplementary Table S1: Cohort definition, diagnostic and intervention codes used in the study.**

| **Assessment** | **Timing** | **Disease, Procedure, or Condition** | **CIHI-DAD, SDS or NACRS ICD-10-CA-CA/ICD-9-CA diagnosis or CCI/CCP procedure codes** | **OHIP ICD-9 diagnosis or fee codes; or other data source** |
| --- | --- | --- | --- | --- |
| **Inclusion criteria** | January 1, 1995 – December 31, 2019 | IKN in RPDB |  | RPDB: field IKN |
| **Exclusion criteria** | Same as above | Missing or nonfemale sex |  | RPDB: field SEX not equal to “F” |
|  | Same as above | No thyroid cancer diagnosis during accrual window. |  | OCR: No record with   - field DXDATE recorded during accrual window, AND - field CURR_TOPOG_CD = “C739” AND - fifth digit of field CURR_MORPH_CD = “3” |
|  | Same as above | Missing birthdate or age <16 or >40 at diagnosis date. |  | RPDB: field BDATE |
|  | Same as above | No cancer treatment initiation within during accrual window or within 2 years of index, or initial cancer treatment of radioactive iodine only. | CIHI-DAD/SDS/NACRS  Less-than-total thyroidectomy:   - CCP: 1910, 1920, 1921, 1922, 1929, 1941, 1942 - CCI: 1FU87   Total thyroidectomy:   - CCP: 1930, 1943 - CCI: 1FU89   Radioactive iodine:   - CCP: 621 - CCI: 1ZZ35, 1FU59 |  |
|  | January 1, 1995 – December 31, 2022 | Death date recorded before index, or within three years of index. |  | RPDB: field DTHDATE |
|  | January 1, 1995 – December 31, 2019 | Missing census subdivision |  | RPDB: field PRCDCSD |
|  | January 1, 1995 – December 31, 2022 | Hysterectomy, BSO, or tubal ligation within 3 years of index. | CIHI-DAD/SDS/NACRS  Hysterectomy:   - CCP:   8030, 8040, 8050, 8060   - CCI: 1RM57, 1RM59, 1RM87, 1RM89, 1RM91, 5MD60CB, 5MD60KE, 5MD60RC, 5MD60RD   BSO:   - CCP: One record of 7740, 7741, 7742, 7750, 7751, 7752   OR  Two records of 7720, 7730   - CCI: Two records of 1RB57, 1RB89, 1RD89   Tubal ligation:   - CCP: 7820, 7821, 7822 - CCI: 1RF51, 1RF59, 1RF89 | Hysterectomy:   - OHIP fee code P042, S757, S758, S759, S763, S810, S816 |
|  | January 1, 1964 – December 31, 2020 | Non-thyroid cancer diagnosis before index or up to 1 year after index |  | OCR:   - field DXDATE recorded before index or up to one year after index,  AND - field CURR_TOPOG_CD not equal to “C739”, AND - fifth digit of field CURR_MORPH_CD = “3” or “6” |
|  | January 1, 1995 – December 31, 2022 | Re-initiation of thyroid cancer treatment after conclusion initial treatment course within 3 years of index. | CIHI-DAD/SDS/NACRS  Less-than-total thyroidectomy:   - CCP: 1910, 1920, 1921, 1922, 1929, 1941, 1942 - CCI: 1FU87   Total thyroidectomy:   - CCP: 1930, 1943 - CCI: 1FU89   Radioactive iodine:   - CCP: 621 - CCI: 1ZZ35, 1FU59 |  |
|  | Same as above | Less than 3 years continuous OHIP coverage after index |  | RPDB |
|  | January 1, 1995 – December 31, 2020 | Occurrence of infertility, early menopause, or POI within one year of index |  | OHIP ICD-9:  Infertility: 628  Early menopause: 627 between the age of 40 and 45  POI: 627 before age 40 |
| **Study exposures** | January 1, 1995 – December 31, 2021 | Initial thyroid cancer treatment | CIHI-DAD/SDS/NACRS  Less-than-total thyroidectomy:   - CCP: 1910, 1920, 1921, 1922, 1929, 1941, 1942 - CCI: 1FU87   Total thyroidectomy:   - CCP: 1930, 1943 - CCI: 1FU89   Radioactive iodine:   - CCP: 621   CCI: 1ZZ35, 1FU59 |  |
| **Main study outcomes** | January 1, 1995 – December 31, 2022 |  |  | OHIP ICD-9:  Infertility: 628  Early menopause: 627 between the age ofs of 40 and 45  POI: 627 before age 40  MOMBABY: live or still birth |
| **Covariates** | January 1, 1995 – December 31, 2019 | Age at index |  | RPDB: field BDATE |
|  | Same as above | Income quintile |  | RPDB: field INCQUINT |
|  | April1, 1988 – December 31, 2019 | Parity |  | MOMBABY: live or still birth recorded prior to index |
|  | January 1, 1985 – December 31, 2019 | Immigration Status |  | IRCC (CIC) |
|  | January 1, 1995 – December 31, 2019 | Rurality |  | RPDB: field RIO2008 |
|  | July 1, 1991 – December 31, 2019 | History of PCOS |  | OHIP ICD-9: 256 |
|  | Same as above | History of endometriosis |  | OHIP ICD-9: 617 |
|  | Same as above | Smoking history |  | OHIP ICD-9: 305 |
|  | Same as above | Obesity |  | OHIP ICD-9: 278 |
|  | January 1, 1993 – December 31, 2019 | HSPN Multimorbidity Indicators  See below for component codes |  |  |
|  |  | Acute Myocardial Infarction | ICD-9-CA: 410  ICD-10-CA: I21 |  |
|  |  | Osteoarthritis | ICD-9-CA: 715  ICD-10-CA: M15-M19 | OHIP ICD-9: 715 |
|  |  | Other Arthritis (includes Synovitis, Fibrositis, Connective tissue disorders, Ankylosing spondylitis, Gout Traumatic arthritis, pyogenic arthritis, Joint derangement, Dupuytren's contracture, Other MSK disorders) | ICD-9-CA: 727, 729, 710, 720, 274, 716, 711, 718, 728, 739  ICD-10-CA: M00-M03, M07, M10, M11-M14, M20-M25, M30-M36, M65-M79 |  |
|  |  | Rheumatoid arthritis | ICD-9-CA: 714  ICD-10-CA: M05-M06 |  |
|  |  | Asthma | ICD-9-CA: 493  ICD-10-CA: J45 |  |
|  |  | Cancer | ICD-9-CA: 140-239  ICD-10-CA: C00-C26, C30-C44, C45-C97 | OHIP ICD-9: 140-239 |
|  |  | Cardiac Arrhythmia | ICD-9-CA: 4273  ICD-10-CA: I480, I481 | OHIP ICD-9: 427 |
|  |  | Congestive Heart Failure | ICD-9-CA: 428  ICD-10-CA: I500, I501, I509 |  |
|  |  | Chronic Obstructive Pulmonary Disease | ICD-9-CA: 491, 492, 496  ICD-10-CA: J41, J42, J43, J44 |  |
|  |  | Coronary syndrome (excluding AMI) | ICD-9-CA: 411-414  ICD-10-CA: I20, I22-I25 | OHIP ICD-9: 411-414 |
|  |  | Dementia | ICD-9-CA: 046.1, 290.0, 290.1, 290.2, 290.3, 290.4, 294, 331.0, 331.1, 331.5  ICD-10-CA: F00, F01, F02, F03, G30 | OHIP ICD-9: 290, 331  ODB |
|  |  | Diabetes | ICD-9-CA: 250  ICD-10-CA: E10, E11, E13, E14 | ODB |
|  |  | Hypertension | ICD-9-CA: 401, 402, 403, 404, 405  ICD-10-CA: I10, I11, I12, I13, I15 |  |
|  |  | (Other) Mental Illnesses | ICD-9-CA: 291, 292, 295, 297, 298, 299, 301, 302, 303, 304, 305, 306, 307, 313, 314, 315, 319  ICD-10-CA: F04, F050, F058, F059, F060, F061, F062, F063, F064, F07, F08, F10, F11, F12, F13, F14, F15, F16, F17, F18, F19, F20, F21, F22, F23, F24, F25, F26, F27, F28, F29, F340, F35, F36, F37, F430, F439, F453, F454, F458, F46, F47, F49, F50, F51, F52, F531, F538, F539, F54, F55, F56, F57, F58, F59, F60, F61, F62, F63, F64, F65, F66, F67, F681, F688, F69, F70, F71, F72, F73, F74, F75, F76, F77, F78, F79, F80, F81, F82, F83, F84, F85, F86, F87, F88, F89, F90, F91, F92, F931, F932, F933, F938, F939, F94, F95, F96, F97, F98 | OHIP ICD-9: 291, 292, 295, 297, 298, 299, 301, 302, 303, 304, 305, 306, 307, 313, 314, 315, 319 |
|  |  | Mood, anxiety, depression and other nonpsychotic disorders | ICD-9-CA: 296, 300, 309, 311  ICD-10-CA: F30, F31, F32, F33, F34 (excl. F34.0), F38, F39, F40, F41, F42, F43.1, F43.2, F43.8, F44, F45.0, F45.1, F45.2, F48, F53.0, F68.0, F93.0, F99 | OHIP ICD-9: 296, 300, 309, 311 |
|  |  | Osteoporosis | ICD-9-CA: 733  ICD-10-CA: M81, M82 | OHIP ICD-9: 733 |
|  |  | Renal failure | ICD-9-CA: 403, 404, 584, 585, 586, V451  ICD-10-CA: N17, N18, N19, T82.4, Z49.2, Z99.2 | OHIP ICD-9: 403, 404, 584, 585, 586, V451 |
|  |  | Stroke (excluding transient ischemic attack) | ICD-9-CA: 430, 431, 432, 434, 436  ICD-10-CA: I60-I64 | OHIP ICD-9: 430, 431, 432, 434, 436 |
